# Impact of pharmacist counseling on reducing instances of adverse events that can affect the quality of life of chemotherapy outpatients with breast Cancer

**DOI:** 10.1186/s40780-018-0105-3

**Published:** 2018-04-30

**Authors:** Kazuhide Tanaka, Akiyo Hori, Tomoya Tachi, Tomohiro Osawa, Katsuhiro Nagaya, Teppei Makino, Seiji Inoue, Masahiro Yasuda, Takashi Mizui, Takumi Nakada, Chitoshi Goto, Hitomi Teramachi

**Affiliations:** 1 Department of Pharmacy, Gifu Municipal Hospital, Gifu, Japan; 2 Laboratory of Clinical Pharmacy, Gifu Pharmaceutical University, Gifu, Japan; 3 Department of Breast Surgery, Gifu Municipal Hospital, Gifu, Japan

**Keywords:** Intervention, Pharmacist, Counseling, Quality of life, Breast cancer, Outpatient, Chemotherapy, Questionnaire, Adverse event

## Abstract

**Background:**

In recent years, cancer chemotherapy is being conducted at outpatient clinics, wherein pharmacists are involved with patient guidance and management of adverse events as experts in medication therapy. Therefore, we clarified the influence of interventions by pharmacists during counseling of patients with cancer on patients’ quality of life.

**Methods:**

To determine this influence, we conducted a survey to assess the quality of life of 39 patients with breast cancer who underwent their initial course of outpatient cancer chemotherapy at Gifu Municipal Hospital. A quality of life survey was conducted before the 1st, 2nd, and 3rd courses of treatment and was based on a method obtained from a survey paper entitled, “Quality of Life Questionnaire for Cancer Patients Treated with Anticancer Drugs.”

**Results:**

Twenty patients were assigned to the intervention group, which received pharmacist counseling, and nineteen patients were assigned to the non-intervention group, which received no pharmacist counseling. Both groups were compared immediately before the 1st course and 2nd course. Regarding the subscale of social relationships, a significant difference was observed for malaise (*p* = 0.043), with the non-intervention group experiencing them to a greater degree than the intervention group. Regarding the change between immediately before the 1st course and the 3rd course, a significant difference was observed in the subscale of social relationships for nausea (*p* = 0.017), with the non-intervention group experiencing it to a greater degree than the intervention group.

**Conclusions:**

The results suggest that receiving pharmacists’ guidance on adverse events and individually adjusted prescriptions tailored to address the occurrence of adverse events improved the treatment environment and enhanced the quality of life in the intervention group. These findings are beneficial in maintaining patients’ quality of life during cancer treatment.

**Trial registration:**

No. UMIN000027171, Registration date: Apr 27, 2017. Retrospectively registered.

## Background

In recent years, cancer chemotherapy treatment has shifted from the inpatient to outpatient setting, and outpatient chemotherapy that involves injections of anticancer drugs during hospital visits is now widely practiced. As developments in supportive therapy and changes in the medical-economics environment have reduced costs, the administration of anticancer drugs does not currently require hospitalization [1, 2]. Anticancer drug therapy has a prominent influence on adverse events caused by chemotherapy, and this has been the subject of several reports [3, 4]. Although the moments when adverse events appear depends on the type of anticancer agent in question, there have been cases where cancer chemotherapy treatment has induced adverse events during the early stages of treatment. Contrary to inpatient cancer chemotherapy, the adverse events experienced by an outpatient receiving cancer chemotherapy can have a direct influence upon home life and work and may cause changes in patients’ quality-of-life (QOL) [5]. Consequently, it is becoming increasingly important to determine the QOL of patients receiving outpatient cancer chemotherapy. Previous reports have stated that outpatients experiencing cancer chemotherapy have a higher QOL compared to those receiving inpatient cancer chemotherapy [6]; however, outpatient cancer chemotherapy involves making the patient responsible for their own prophylaxis and responses to adverse events and, if they do not correctly administer treatment for the events, there is a risk that the events will worsen. Before beginning outpatient cancer chemotherapy, it is necessary that medical staff provide patients with a sufficient explanation of the treatment details, as this will give them important information concerning expected adverse events related to their chemotherapy and effective treatments for these events. The medical staff should also confirm that their instructions are followed; this way, it may be possible to minimize the impact of the adverse events [7]. In addition, to accurately evaluate the adverse events that can occur in home situations once outpatient cancer chemotherapy commences, it is very important to take measures relating to planning, such as dose adjustments, and provide supportive therapy in the subsequent treatments [8]. An attending physician cannot provide guidance and manage the adverse events of all their patients; therefore, this service is performed by medical staff such as pharmacists. This provision of supportive medical care can reduce the burden on attending physicians while also minimizing patients’ difficulties related to adverse events.

Numerous pharmacist intervention assessments have been performed on outpatients regarding various diseases, including cancer chemotherapy studies involving pharmacists have investigated the proper use of oral anti-cancer agents [9], cost improvement through the proper use of antiemetic agents [10, 11], cost avoidance through regimen reviews and patient counseling [12], pharmaceutical care as a means of reducing the psychological burden on patients [13], reductions in the occurrence of adverse events because of prescription proposals from pharmacists [14], and the key role of disease management and supportive-care management [15]. And numerous QOL assessments have been performed regarding breast cancer. There have also been investigations into QOL before-and-after a breast cancer diagnosis [16], QOL after both breast cancer diagnosis and survival [17], QOL and preferences for treatment following systemic adjuvant therapy [18], QOL following surgery [19], the impact of surgery and chemotherapy on QOL [20], adverse effects of chemotherapy and QOL [21], and the effect chemotherapy has on QOL and presenteeism [22]. In this way, there are many reports about interventions with patients experiencing outpatient chemotherapy provided by pharmacists, and QOL assessments of patients with breast cancer in various situations; however, to our knowledge, no reports have examined the relevance of both pharmacists’ intervention and patients’ QOL. Therefore, we investigated the influence of adverse event change by pharmacist counseling on the QOL of outpatients receiving breast cancer chemotherapy.

## Methods

### Participants and treatment period

Participants were 39 patients with breast cancer who received their first course of intravenous chemotherapy at Gifu Municipal Hospital between December 2013 and November 2015. None of the patients received hormone therapy, only oral anticancer drug treatment or radiation therapy. All patients received appropriate.

Supportive therapy that conformed with the chemotherapy treatment guidelines for patients with breast cancer. We asked the participants to answer self-completed survey questionnaires before their 1st, 2nd, and 3rd courses of outpatient chemotherapy (day 1 of the 1st, 2nd, and 3rd courses, respectively).

### Mandatory intervention

While this study was ongoing, the hospital created a policy to provide all patients with cancer with personal counseling from a pharmacist before each medical examination. After the introduction of this personal counseling, the pharmacist provided counseling to all breast cancer outpatient chemotherapy patients before their medical examinations, irrespective of whether the patients were participating. To navigate this serious obstacle, patients who participated before personal counseling was made mandatory were retrospectively assigned to the non-intervention group, and those who received counseling because of this new policy were retrospectively assigned to the intervention group. Patients who were initially treated at the Outpatient Chemotherapy Center in our hospital received, before their first treatment began, guidance concerning the treatment that would be provided by the doctor, pharmacist, and nurse. Therefore, even patients in the non-intervention group received guidance on their treatment from the doctor, pharmacist, and nurse, similar to the other chemotherapy outpatients.

### Pharmacist intervention

The pharmacist provided personal counseling for each patient immediately before the medical examination, which was conducted on the day of patient received outpatient cancer chemotherapy. The pharmacist performed the counseling in accordance with patients’ condition and their degree of understanding; however, the following counseling and guidance content was common to all patients: the counseling before the 1st course concerned corrective action for expected adverse events and the correct method of taking the prescribed medicine used for supportive therapy. The counseling before the 2nd and 3rd courses concerned situations in which adverse events occurred and providing guidance on methods of treating these adverse events. The pharmacist who performed the personal counseling before the medical examinations had over five years of experience in cancer chemotherapy and had completed the appropriate training relating to cancer treatment; they also had a sufficient record of accomplishmen regarding to providing drug-related advice for cancer patients and operating as a primary contact. In instances where the main pharmacist was absent, the secondary pharmacist responded.

### Survey items

The items surveyed in this study were patient attributes, QOL, and adverse events. Patient attributes obtained from the survey comprised marital status, the presence of cohabitants, and current occupation. Data were also obtained from patients’ electronic medical records including age; stage classification of breast cancer progression; performance status (PS); human epidermal growth factor receptor type 2 (HER2), estrogen receptor (ER), and progesterone receptor (PgR) status; purpose of chemotherapy (adjuvant, neoadjuvant); and treatment regimen. QOL was assessed using the Quality of Life Questionnaire for Cancer Patients Treated with Anticancer Drugs (QOL-ACD) [23]. The QOL-ACD is a cancer-specific scale and its reliability and validity have been confirmed among patients with cancer in Japan. Furthermore, QOL assessments using the QOL-ACD have also been previously used in relation to patients with breast cancer [19, 24, 25]. The QOL-ACD comprises four subscales (activity, six items; physical condition, six items; psychological condition, five items; and social relationships, five items) and a face scale (one item) for measuring overall QOL (a total of 23 items). The lowest QOL for each item is given a score of 1, whereas the highest QOL is given a score of 5; thus, the QOL-ACD gives a minimum total score of 23 points and maximum total score of 115 points.

Information regarding the occurrence of adverse events before the 1st, 2nd, and 3rd courses of outpatient chemotherapy was obtained from patients’ electronic medical records. Adverse event items were identified by consulting expert nurses from the Outpatient Chemotherapy Center and these were evaluated based on the Common Terminology Criteria for Adverse Events, version4.0 (CTCAE). A grade of 0 indicated no adverse events, whereas grade 1 or higher indicated the presence of adverse events.

### Analysis and statistical processing

The total score for QOL-ACD and the mean score for each QOL-ACD subscale were compared between the non-intervention group and the intervention group. Both scores were measured immediately before the 2nd and 3rd courses, and these results were then compared with the equivalent scores taken immediately before the 1st course of chemotherapy. In addition, changes in QOL after chemotherapy were compared between the patient groups and stratified according to the occurrence of individual adverse events in both groups.

The IBM Statistical Package for Social Science (SPSS) Statistics Version 22.0 software was used for statistical processing.

Wilcoxon signed-rank test was used to test for differences between the groups before and after chemotherapy regarding the total score for QOL-ACD and the mean score for each QOL-ACD subscale. Mann-Whitney’s U-test was used to conduct intergroup comparisons of the total QOL-ACD score and the mean score for each subscale before the 1st, 2nd, and 3rd courses, and the differences between the patient groups were stratified according to the occurrence of individual adverse events. Fisher’s exact test was used to perform an intergroup comparison of patient attributes regarding the occurrence of individual adverse events. *P*-value of < 0.05 was considered to be statistically significant.

### Ethics statement

This study was approved by the Ethical Review Board of Gifu Municipal Hospital. (approval no. 186). Participants were given sufficient explanation of the study in writing including the following contents: purpose of the study, research method, subject of study, cost, ethical consideration, and management of personal information. After the explanation, participants provided written, informed consent. Regarding ethical considerations for the non-intervention group, personal counseling with a pharmacist was not provided; however, the pharmacist and other medical staff could respond to questions and engage in consultation concerning the therapeutic agents and adverse events involved in treatment.

## Results

All results are expressed in the following format: the non-intervention group and, the intervention group.

### Patient attributes

Patient attributes are shown in Table 1. All patients were women (mean ages 53.3 years and 56.3 years, respectively). All patients had a PS of 0, and most patients in the two groups (44.4% and, 52.4%, respectively) had stage-2 cancer. HER2 expression was positive for 26.3%, 23.8%, respectively; ER was positive for 77.8%, 71.4%, respectively; and PgR was positive for 55.6%, 52.4%, respectively. The purpose of chemotherapy was 61.1% and 40.0% neoadjuvant therapy, respectively and 38.9% and 60.0% adjuvant therapy, respectively. Most patients (61.1% and, 55.0%, respectively) were administered a regimen of epirubicin plus cyclophosphamide. In addition, 68.4% and 80.0% of the patients were married, respectively, and 100% and 95.0% of the patients lived with another individual, respectively. There was no significant difference between the Non-intervention group and the intervention group regarding any patient-attribute item (*p* > 0.05).

**Table 1:** Patient attributes

Item	Non intervention	Intervention	*P*
	n (%)	n (%)	
Age (year)			
Mean ± Standard deviation	53.3 ± 11.1	56.3 ± 9.0	0.313
Gender			
Female	19 (100)	20 (100)	1.000
Male	0 (0)	0 (0)	
PS			
0	19 (100)	20 (100)	1.000
1	0 (0)	0 (0)	
2	0 (0)	0 (0)	
Stage			
I	3 (16.7)	8 (38.1)	0.238
II	8 (44.4)	11 (52.4)	
III	5 (22.2)	2 (9.5)	
IV	3 (16.7)	0 (0)	
HER2			
0	1 (5.6)	1 (4.8)	0.213
+ 1	9 (44.4)	13 (61.9)	
+ 2	6 (33.3)	2 (9.5)	
FISH-	4	2	
FISH+	2		
+ 3	3 (16.7)	5 (23.8)	
ER			
+	15 (77.8)	15 (71.4)	0.223
±	2 (11.1)	4 (19.0)	
–	2 (11.1)	2 (9.5)	
PgR			
+	10 (55.6)	11 (52.4)	0.223
±	4 (16.7)	5 (23.8)	
–	5 (27.8)	5 (23.8)	
Purpose of chemotherapy			
Neoadjuvant	11 (61.1)	8 (40.0)	0.343
Adjuvant	8 (38.9)	12 (60.0)	
Regimen			
Anthracyclines			0.110
EC (every 3w)	11 (61.1)	11 (55.0)	
FEC (every 3w)	0 (0)	3 (15.0)	
Taxanes			
TC (every 3w)	3 (16.7)	6 (30.0)	
nabPTX (every 3w)	1 (5.6)	0 (0)	
PTX + BV (every 4w)	1 (5.6)	0 (0)	
Others			
CMF (every 3w)	3 (16.7)	0 (0)	
Marital status			
Married	13 (68.4)	16 (80.0)	0.480
Unmarried	6 (31.6)	4 (20.0)	
Cohabitants			
Yes	19 (100)	19 (95.0)	1.000
No	0 (0)	1 (5.0)	
Current occupation			
Employer	12 (63.2)	9 (45.0)	0.341
Non-employee	7 (36.8)	11 (55.0)	

Fisher’s exact test, *PS* performance status, *HER2* human epidermal growth factor receptor type 2, *ER* estrogen receptor, *PgR* progesterone receptor, *EC* epirubicin/cyclophosphamide, *FEC* fluorouracil/epirubicin/cyclophosphamide, *TC* docetaxel/cyclophosphamide, *nab-PTX* nanoparticle albumin-bound paclitaxel, *PTX* paclitaxel, *BV* Bevacizumab, *CMF* cyclophosphamide/methotrexate/fluorouracil

### Occurrence of adverse events

Occurrence of adverse events is shown in Table 2. No grade-4 adverse events were observed during the study period. Before the 1st course, no grade 2 or 3 adverse events were observed in any item. Certain adverse events occurred in over half of the patients before the 2nd course. The non-intervention group experienced nausea (52.7%), and both groups experienced malaise (63.2% and 50.0%, respectively), anorexia (52.6% and 55.0%, respectively), and alopecia (89.5% and 95.0%, respectively). Regarding adverse items that occurred in over 50% of patients before the 3rd course, the intervention group experienced constipation (90.0%) and both groups experienced malaise (63.2% and 55.0%, respectively), anorexia (63.2% and 50.0%, respectively), alopecia (100% and 95.0%, respectively), and nail ridging (57.9% and 65.0%, respectively). No statistically significant difference existed between the two groups for any adverse event item at any point.

**Table 2:** Occurrence of individual adverse events in both groups

Adverse events n (%)		Before 1st course			Before 2nd course			Before 3rd course		
	Grade	Non intervention	Intervention	*P*	Non intervention	Intervention	*P*	Non intervention	Intervention	*P*
Constipation	2	0 (0)	0 (0)	0.342	2 (10.5)	2 (10.0)	0.975	3 (15.8)	3 (15.0)	0.975
	1	3 (15.8)	1 (5.0)		6 (31.6)	7 (35.0)		6 (31.6)	7 (35.0)	
	0	16 (84.2)	19 (95.0)		11 (57.9)	11 (55.0)		10 (52.6)	10 (50.0)	
Nausea	2	0 (0)	0 (0)	1.000	1 (5.3)	0 (0)	0.264	0 (0)	0 (0)	0.155
	1	1 (5.3)	1 (5.0)		9 (47.4)	6 (30.0)		7 (36.8)	3 (15.0)	
	0	18 (94.7)	19 (95.0)		9 (47.4)	14 (70.0)		12 (63.2)	17 (85.0)	
Oral pain	2	0 (0)	0 (0)	1.000	0 (0)	0 (0)	0.523	0 (0)	1 (5.0)	0.490
	1	0 (0)	0 (0)		9 (47.4)	7 (35.0)		6 (31.6)	8 (40.0)	
	0	19 (100)	20(100)		10 (52.6)	13 (65.0)		13 (68.4)	11 (55.0)	
Vomiting	2	0 (0)	0 (0)	1.000	0(0)	1 (5.0)	0.126	0 (0)	0 (0)	0.342
	1	0 (0)	0 (0)		5 (26.3)	1 (5.0)		3 (15.8)	1 (5.0)	
	0	19 (100)	20 (100)		14 (73.7)	18 (90.0)		16 (84.2)	19 (95.0)	
Fever	2	0 (0)	0 (0)	1.000	2 (10.5)	1 (5.0)	0.443	0 (0)	0 (0)	0.605
	1	0 (0)	0 (0)		2 (10.5)	5 (25.0)		1 (5.3)	3 (15.0)	
	0	19 (100)	20 (100)		15 (78.9)	14 (70.0)		18 (94.7)	17 (85.0)	
Malaise	2	0 (0)	0 (0)	0.342	4 (21.1)	3 (15.0)	0.700	1 (5.3)	2 (10.0)	0.684
	1	3 (15.8)	1 (5.0)		8 (42.1)	7 (35.0)		11 (57.9)	9 (45.0)	
	0	16 (84.2)	19 (95.0)		7 (36.8)	10 (50.0)		7 (36.8)	9 (45.0)	
Anorexia	2	0 (0)	0 (0)	0.231	3 (15.8)	2 (10.0)	0.809	1 (5.3)	1 (5.0)	0.703
	1	2 (10.5)	0 (0)		7 (36.8)	9 (45.0)		11 (57.9)	9 (45.0)	
	0	17 (89.5)	20 (100)		9 (47.4)	9 (45.0)		7 (36.8)	10 (50.0)	
Peripheral sensory neuropathy	2	0 (0)	0 (0)	1.000	0 (0)	0 (0)	0.695	0 (0)	0 (0)	1.000
	1	1 (5.3)	1 (5.0)		3 (15.8)	5 (25.0)		4 (21.1)	4 (20.0)	
	0	18 (94.7)	19 (95.0)		16 (84.2)	15 (75.0)		15 (78.9)	16 (80.0)	
Alopecia	2	1 (5.3)	0 (0)	0.487	14 (73.7)	11 (55.0)	0.230	16 (84.2)	17 (85.0)	0.547
	1	0 (0)	0 (0)		3 (15.8)	8 (40.0)		3 (15.8)	2 (10.0)	
	0	18 (94.7)	20 (100)		2 (10.5)	1 (5.0)		0 (0)	1 (5.0)	
Nail ridging	2	0 (0)	0 (0)	1.000	0 (0)	0 (0)	1.000	0 (0)	0 (0)	0.748
	1	2 (10.5)	3 (15.0)		3 (15.8)	4 (20.0)		11 (57.9)	13 (65.0)	
	0	17 (89.5)	17 (85.0)		16 (84.2)	16 (80.0)		8 (42.1)	7 (35.0)	
Skin disorder	2	0 (0)	0 (0)	0.342	1 (5.3)	0 (0)	0.561	1 (5.3)	0 (0)	0.581
	1	3 (15.8)	1 (5.0)		8 (42.1)	8 (40.0)		7 (36.8)	8 (40.0)	
	0	16 (84.2)	19 (95.0)		10 (52.6)	12 (60.0)		11 (57.9)	12 (60.0)	

Fisher’s exact test

### QOL assessment

QOL changes in QOL-ACD are shown in Table 3. There was no statistically significant difference in the intergroup comparisons regarding the total QOL-ACD score or the mean for each subscale before the 1st, 2nd, or 3rd courses. Regarding the mean scores for each QOL-ACD subscale, the main difference between the status before the 2nd course and that before the 1st course was a significant decrease in activity in both groups (3.53 ±5.33; *p* = 0.011 and 3.05 ±3.41; *p* = 0.003, respectively); furthermore, physical condition significantly decreased in the intervention group (1.75 ±2.85; *p* = 0.017) and social relationships significantly increased in both groups (−2.16 ±2.97; *p* = 0.009 and −3.50 ±4.50; *p* = 0.003, respectively). Regarding the differences between before the 3rd course and before the 1st course in relation to the mean scores for each QOL-ACD subscale, activity (2.42 ±4.36; *p* = 0.021 and 3.25 ±3.61; *p* = 0.002, respectively) significantly decreased in both groups, physical condition significantly decreased in the intervention group (1.75 ±3.15; *p* = 0.027), and social relationships significantly increased in both groups (−1.84 ±3.11; *p* = 0.019 and −2.55 ±4.42; *p* = 0.018, respectively).

**Table 3:** Comparison of the QOL-ACD total score and mean score for each subscale before each courses in both groups

			Before 1st course		Before 2nd course		Before 3rd course		The difference between 1st course			
	Existence of intervention	n	(A)	*P*	(B)	*P*	(C)	*P*	(B) - (A)	*P*	(C) - (A)	*P*
Total score	Non intervention	19	88.16 ± 13.40	0.396	84.58 ± 16.43	0.380	85.26 ± 15.17	0.569	3.58 ± 10.73	0.163	2.90 ± 7.49	0.087
	Intervention	20	91.55 ± 9.69		89.60 ± 11.45		88.45 ± 10.85		1.95 ± 8.22	0.302	3.10 ± 7.53	0.100
Mean score of subscales												
Activity	Non Intervention	19	4.61 ± 0.66	0.607	4.03 ± 0.96	0.351	4.21 ± 0.77	0.879	3.53 ± 5.33	0.011*	2.42 ± 4.36	0.021*
	Intervention	20	4.80 ± 0.57		4.29 ± 0.76		4.26 ± 0.69		3.05 ± 3.41	0.003*	3.25 ± 3.61	0.002*
Physical condition	Non intervention	19	4.33 ± 0.69	0.857	4.03 ± 0.79	0.879	4.07 ± 0.74	0.792	1.84 ± 4.40	0.066	1.58 ± 3.15	0.086
	Intervention	20	4.43 ± 0.50		4.14 ± 0.50		4.15 ± 0.49		1.75 ± 2.85	0.017*	1.70 ± 3.15	0.027*
Psychological condition	Non intervention	19	3.94 ± 0.67	0.647	3.81 ± 0.83	0.607	3.75 ± 0.87	0.647	0.63 ± 2.48	0.466	0.95 ± 2.76	0.267
	Intervention	20	4.05 ± 0.64		3.92 ± 0.77		3.89 ± 0.63		0.65 ± 3.57	0.568	0.80 ± 3.29	0.362
Social relationships	Non intervention	19	2.32 ± 0.91	0.496	2.75 ± 0.73	0.057	2.68 ± 0.71	0.184	−2.16 ± 2.97	0.009*	−1.84 ± 3.11	0.023*
	Intervention	20	2.45 ± 0.83		3.15 ± 0.62		2.96 ± 0.69		−3.50 ± 4.50	0.003	−2.55 ± 4.42	0.025

Mann-Whitney’s U-test: Comparison of both groups in each course, Wilcoxon signed-rank test: Comparison of scores within group, * *P* < 0.05, mean ± standard deviation

### Relationship between QOL and the occurrence of adverse events

Tables 4 and 5 show the QOL-ACD results concerning the occurrence of individual adverse events in the Non-intervention group and the intervention group.

**Table 4:** Comparison of the QOL-ACD total score and mean score for each subscale before 1st and 2nd course in patients with adverse events of both groups

			Total score				Subscale															
Adverse event	Existence of intervention	n	Before 1st course	Before 2nd course	Difference	*P*	Activity			*P*	Physical condition			*P*	Psychological condition			*P*	Social relationships			*P*
							Before 1st course	Before 2nd course	Difference		Before 1st course	Before 2nd course	Difference		Before 1st course	Before 2nd course	Difference		Before 1st course	Before 2nd course	Difference	
			(A)	(B)	(B-A)		(A)	(B)	(B-A)		(A)	(B)	(B-A)		(A)	(B)	(B-A)		(A)	(B)	(B-A)	
Constipation	Non intervention	8	87.75 ± 12.93	77.63 ± 19.40	−10.13 ± 13.10	0.481	4.67 ± 0.59	3.52 ± 1.16	−1.15 ± 1.01	0.277	4.40 ± 0.62	3.67 ± 0.86	−0.73 ± 0.58	0.321	4.00 ± 0.66	3.50 ± 0.90	− 0.50 ± 0.55	0.606	2.03 ± 0.96	2.75 ± 0.76	0.73 ± 0.63	0.200
	Intervention	9	92.56 ± 12.37	87.56 ± 13.67	−5.00 ± 7.09		4.70 ± 0.83	4.15 ± 0.92	−0.56 ± 0.54		4.50 ± 0.38	4.11 ± 0.24	− 0.39 ± 0.37		4.07 ± 0.77	3.78 ± 1.00	− 0.29 ± 0.90		2.69 ± 0.68	3.04 ± 0.67	0.36 ± 0.42	
Nausea	Non intervention	10	83.20 ± 13.18	77.70 ± 14.60	−5.50 ± 10.78	0.875	4.43 ± 0.79	3.72 ± 0.99	− 0.72 ± 0.82	1.000	4.10 ± 0.81	3.65 ± 0.70	− 0.45 ± 0.73	0.313	3.84 ± 0.65	3.56 ± 0.81	− 0.28 ± 0.51	1.000	2.00 ± 0.71	2.50 ± 0.58	0.50 ± 0.65	0.263
	Intervention	6	91.17 ± 5.67	87.67 ± 13.10	−3.50 ± 11.57		5.00 ± 0.00	4.22 ± 0.75	− 0.78 ± 0.75		4.33 ± 0.71	4.00 ± 0.81	− 0.33 ± 0.26		4.20 ± 0.62	3.90 ± 0.83	− 0.30 ± 1.02		2.07 ± 0.63	3.07 ± 0.69	1.00 ± 0.89	
Oral pain	Non intervention	9	87.33 ± 15.57	83.89 ± 17.50	−3.44 ± 7.96	0.408	4.52 ± 0.71	3.94 ± 3.94	− 0.57 ± 0.69	0.351	4.24 ± 0.94	3.93 ± 0.76	− 0.31 ± 0.68	0.758	4.02 ± 0.76	3.80 ± 1.02	− 0.22 ± 0.39	0.918	2.33 ± 0.85	2.82 ± 0.61	0.49 ± 0.67	1.000
	Intervention	7	95.29 ± 6.18	89.14 ± 7.69	−6.14 ± 7.82		4.88 ± 0.25	4.05 ± 0.57	− 0.83 ± 0.54		4.33 ± 0.46	4.07 ± 0.46	− 0.26 ± 0.53		4.23 ± 0.45	3.97 0.42	−0.26 ± 0.70		3.00 ± 0.42	3.40 ± 0.54	0.40 ± 0.74	
Vomiting	Non intervention	5	85.60 ± 7.80	78.20 ± 10.62	−7.40 ± 7.67	0.571	4.53 ± 0.73	3.93 ± 0.80	−0.60 ± 0.91	1.000	4.40 ± 0.30	3.60 ± 0.48	− 0.80 ± 0.22	0.190	3.84 ± 0.61	3.48 ± 0.92	− 0.36 ± 0.41	0.571	2.00 ± 0.57	2.36 ± 0.46	0.36 ± 0.61	0.095
	Intervention	2	93.00 ± 2.83	94.50 ± 16.26	1.50 ± 13.44		5.00 ± 0.00	4.33 ± 0.94	−0.67 ± 0.94		4.50 ± 0.71	4.08 ± 1.06	− 0.42 ± 0.35		4.40 ± 0.85	4.30 ± 0.99	− 0.10 ± 0.14		1.90 ± 1.27	3.90 ± 0.42	2.00 ± 0.85	
Fever	Non intervention	4	94.50 ± 7.94	97.50 ± 7.51	3.00 ± 2.94	0.114	5.00 ± 0.00	4.92 ± 0.10	−0.08 ± 0.10	0.257	4.75 ± 0.29	4.50 ± 0.41	− 0.25 ± 0.17	0.352	4.40 ± 0.54	4.60 ± 0.43	0.20 ± 0.23	0.476	2.05 ± 0.76	2.85 ± 0.64	0.80 ± 0.67	0.476
	Intervention	6	89.83 ± 14.36	87.17 ± 16.08	−2.67 ± 5.43		4.53 ± 1.00	4.03 ± 1.04	−0.50 ± 0.45		4.58 ± 0.36	4.03 ± 0.32	− 0.56 ± 0.43		3.90 ± 0.75	3.87 ± 1.19	− 0.03 ± 0.69		2.43 ± 0.78	3.07 ± 0.64	0.63 ± 0.46	
Malaise	Non intervention	12	87.42 ± 11.40	79.58 ± 16.00	−7.83 ± 10.90	0.069	4.64 ± 0.64	3.78 ± 1.02	−0.86 ± 0.95	0.254	4.39 ± 0.50	3.72 ± 0.76	− 0.67 ± 0.54	0.159	4.02 ± 0.55	3.72 ± 0.82	− 0.30 ± 0.54	0.203	2.03 ± 0.85	2.53 ± 0.67	0.50 ± 0.66	0.043*
	Intervention	10	86.00 ± 8.83	88.10 ± 13.88	2.10 ± 7.97		4.67 ± 0.78	4.23 ± 0.95	−0.43 ± 0.64		4.40 ± 0.55	4.07 ± 0.66	−0.33 ± 0.41		3.74 ± 0.56	3.84 ± 0.95	0.10 ± 0.63		1.86 ± 0.65	3.10 ± 0.57	1.24 ± 0.85	
Anorexia	Non intervention	10	81.60 ± 11.51	73.30 ± 12.62	−8.30 ± 12.00	0.152	4.43 ± 0.79	3.48 ± 1.02	−0.95 ± 1.01	0.152	4.08 ± 0.79	3.45 ± 0.60	−0.63 ± 0.83	0.099	3.76 ± 0.59	3.36 ± 0.71	− 0.40 ± 0.52	0.099	1.80 ± 0.60	2.38 ± 0.44	0.58 ± 0.69	0.468
	Intervention	11	90.64 ± 8.16	90.82 ± 9.02	0.18 ± 7.31		4.92 ± 0.16	4.47 ± 0.60	−0.45 ± 0.63		4.39 ± 0.54	4.09 ± 0.60	− 0.30 ± 0.45		3.84 ± 0.56	3.95 ± 0.51	0.11 ± 0.64		2.36 ± 0.79	3.22 ± 0.60	0.85 ± 0.82	
Peripheral sensory neuropathy	Non intervention	3	80.67 ± 8.39	75.33 ± 8.74	−5.33 ± 17.01	0.571	4.33 ± 0.67	3.61 ± 0.77	−0.72 ± 1.39	0.571	4.22 ± 0.42	4.00 ± 0.83	− 0.22 ± 1.07	1.000	3.20 ± 0.53	2.73 ± 0.46	− 0.47 ± 0.58	0.571	2.07 ± 0.50	2.47 ± 0.46	0.40 ± 0.20	0.250
	Intervention	5	85.60 ± 12.38	82.80 ± 14.34	−2.80 ± 6.98		4.43 ± 1.08	3.90 ± 1.07	−0.53 ± 0.40		4.43 ± 0.15	4.03 ± 0.46	−0.40 ± 0.52		3.64 ± 0.50	3.44 ± 1.03	− 0.20 ± 0.60		2.20 ± 1.01	2.88 ± 0.33	0.68 ± 1.01	
Alopecia	Non intervention	17	90.06 ± 12.86	85.35 ± 17.08	−4.71 ± 10.42	0.876	4.75 ± 0.56	4.01 ± 1.01	−0.74 ± 0.81	0.616	4.38 ± 0.71	4.04 ± 0.77	−0.34 ± 0.68	0.925	4.02 ± 0.64	3.87 ± 0.86	− 0.15 ± 0.52	0.851	2.37 ± 0.95	2.84 ± 0.72	0.47 ± 0.62	0.573
	Intervention	19	91.74 ± 9.92	89.26 ± 11.67	−2.47 ± 8.09		4.79 ± 0.58	4.25 ± 0.76	−0.54 ± 0.57		4.43 ± 0.51	4.11 ± 0.50	−0.32 ± 0.47		4.05 ± 0.66	3.94 ± 0.79	− 0.12 ± 0.73		2.50 ± 0.83	3.15 ± 0.64	0.65 ± 0.90	
Nail ridging	Non intervention	3	93.67 ± 9.29	87.00 ± 17.06	−6.67 ± 10.02	0.629	4.89 ± 0.19	4.22 ± 0.92	−0.67 ± 1.01	0.857	4.50 ± 0.33	3.89 ± 0.67	−0.61 ± 0.35	1.000	4.13 ± 0.99	3.80 ± 1.39	− 0.33 ± 0.42	0.229	2.60 ± 1.20	3.13 ± 0.61	0.53 ± 0.61	0.400
	Intervention	4	92.75 ± 9.74	92.00 ± 11.46	−0.75 ± 5.74		4.75 ± 0.29	4.13 ± 0.60	− 0.63 ± 0.44		4.42 ± 0.57	4.13 ± 0.44	− 0.29 ± 0.90		4.10 ± 0.62	4.15 0.74	0.05 ± 0.34		2.65 ± 0.77	3.50 ± 0.62	0.85 ± 0.25	
Skin disorder	Non intervention	9	91.33 ± 14.26	84.56 ± 19.74	−6.78 ± 10.27	0.606	4.70 ± 0.56	3.89 ± 1.10	− 0.81 ± 0.83	0.606	4.44 ± 0.66	3.98 ± 0.89	− 0.46 ± 0.41	0.815	4.11 ± 0.71	3.80 ± 0.97	− 0.31 ± 0.53	0.743	2.44 ± 1.00	2.91 ± 0.82	0.47 ± 0.63	0.321
	Intervention	8	90.00 ± 8.65	87.63 ± 9.61	−2.38 ± 10.32		4.83 ± 0.27	4.25 ± 0.70	− 0.58 ± 0.73		4.27 ± 0.59	3.98 ± 0.58	− 0.29 ± 0.60		4.00 ± 0.57	3.75 ± 0.44	− 0.25 ± 0.67		2.35 ± 0.95	3.18 ± 0.50	0.83 ± 1.05	

Mann-Whitney's U-test, mean±standard deviation, * *P* < 0.05

**Table 5:** Comparison of the QOL-ACD total score and mean score for each subscale before 1st and 3rd course in patients with adverse events of both groups

			Total score				Subscale															
Adverse event	Existence of intervention	n	Before 1st course	Before 3rd course	Difference	*P*	Activity			*P*	Physical condition			*P*	Psychological condition			*P*	Social relationships			*P*
							Before 1st course	Before 3rd course	Difference		Before 1st course	Before 3rd course	Difference		Before 1st course	Before 3rd course	Difference		Before 1st course	Before 3rd course	Difference	
			(A)	(C)	(C-A)		(A)	(C)	(C-A)		(A)	(C)	(C-A)		(A)	(C)	(C-A)		(A)	(C)	(C-A)	
Constipation	Non intervention	9	84.67 ± 16.19	82.56 ± 18.60	−2.11 ± 5.58	0.604	4.33 ± 0.78	4.04 ± 0.88	−0.30 ± 0.52	0.447	4.20 ± 0.93	3.93 ± 0.91	− 0.28 ± 0.57	0.905	3.93 ± 0.67	3.64 ± 1.03	− 0.26 ± 0.54	0.604	2.16 ± 0.98	2.67 ± 0.70	0.51 ± 0.76	0.661
	Intervention	10	91.10 ± 11.46	87.50 ± 10.87	−3.60 ± 7.43		4.67 ± 0.78	4.20 ± 0.68	−0.47 ± 0.55		4.32 ± 0.41	4.13 ± 0.31	−0.18 ± 0.40		4.00 ± 0.72	3.74 ± 0.63	− 0.26 ± 0.74		2.70 ± 0.64	3.00 ± 0.84	0.30 ± 0.65	
Nausea	Non intervention	7	86.00 ± 6.95	80.57 ± 12.03	−5.43 ± 10.97	0.117	4.67 ± 0.64	4.12 ± 0.74	− 0.55 ± 0.74	1.000	4.38 ± 0.28	3.86 ± 0.75	− 0.52 ± 0.68	0.183	3.94 ± 0.54	3.49 ± 0.78	− 0.46 ± 0.67	0.017*	1.77 ± 0.41	2.37 ± 0.39	0.60 ± 0.52	0.383
	Intervention	3	88.00 ± 7.00	93.33 ± 10.26	5.33 ± 3.51		5.00 ± 0.00	4.44 ± 0.69	−0.56 ± 0.69		4.06 ± 0.92	4.17 ± 0.76	0.11 ± 0.25		3.67 ± 0.23	4.27 ± 0.12	0.60 ± 0.20		2.27 ± 0.46	3.20 ± 0.20	0.93 ± 0.31	
Oral pain	Non intervention	6	82.50 ± 12.34	77.00 ± 13.78	−5.50 ± 7.04	0.776	4.44 ± 0.86	3.86 ± 0.92	−0.58 ± 0.57	0.689	4.14 ± 0.61	3.75 ± 0.74	−0.39 ± 0.75	0.529	3.80 ± 0.56	3.33 ± 0.91	− 0.50 ± 0.70	0.689	1.90 ± 0.64	2.37 ± 0.32	0.47 ± 0.74	0.529
	Intervention	9	94.44 ± 8.63	88.44 ± 11.77	−6.00 ± 9.17		4.96 ± 0.07	4.17 ± 0.76	−0.80 ± 0.72		4.54 ± 0.59	4.09 ± 0.51	−0.44 ± 0.35		4.20 ± 0.56	3.84 ± 0.73	− 0.36 ± 0.83		2.53 ± 0.69	3.16 ± 0.49	0.62 ± 0.54	
Vomiting	Non intervention	3	85.67 ± 11.02	77.67 ± 16.50	−8.00 ± 9.64	0.500	4.44 ± 0.96	4.33 ± 0.73	−0.11 ± 0.25	0.500	4.39 ± 0.35	3.44 ± 0.84	− 0.94 ± 0.67	0.500	4.13 ± 0.50	3.40 ± 1.25	− 0.73 ± 0.81	0.500	1.93 ± 0.61	2.27 ± 0.42	0.33 ± 0.58	1.000
	Intervention	1	91.00 ± 0	96.00 ± 0	5.00 ± 0		5.00 ± 0	4.67 ± 0	− 0.33 ± 0		4.00 ± 0	4.33 ± 0	0.33 ± 0		3.80 ± 0	4.20 ± 0	0.40 ± 0		2.80 ± 0	3.40 ± 0	0.60 ± 0	
Fever	Non intervention	1	85.00 ± 0	86.00 ± 0	1.00 ± 0	1.000	4.33 ± 0	3.83 ± 0	−0.50 ± 0	1.000	4.67 ± 0	4.50 ± 0	−0.17 ± 0	0.500	3.80 ± 0	3.60 ± 0	− 0.20 ± 0	1.000	1.60 ± 0	2.80 ± 0	1.20 ± 0	0.500
	Intervention	3	90.67 ± 4.04	87.00 ± 17.35	−3.67 ± 13.58		4.94 ± 0.10	4.28 ± 1.25	−0.67 ± 1.15		4.78 ± 0.19	4.22 ± 0.59	− 0.56 ± 0.42		4.00 ± 0.53	3.53 ± 0.90	− 0.47 ± 0.92		1.87 ± 0.23	2.93 ± 0.31	1.07 ± 0.12	
Malaise	Non intervention	12	86.75 ± 13.38	82.33 ± 14.72	−4.42 ± 7.81	0.413	4.00 ± 0.81	4.00 ± 0.81	− 0.64 ± 0.67	0.928	4.00 ± 0.75	4.00 ± 0.75	− 0.25 ± 0.56	0.487	3.60 ± 0.85	3.60 ± 0.85	− 0.28 ± 0.55	0.069	2.60 ± 0.57	2.60 ± 0.57	0.42 ± 0.73	0.786
	Intervention	11	88.36 ± 5.14	86.91 ± 9.53	−1.45 ± 7.48		4.21 ± 0.69	4.21 ± 0.69	−0.65 ± 0.65		4.08 ± 0.55	4.08 ± 0.55	−0.17 ± 0.67		3.80 ± 0.55	3.80 ± 0.55	0.11 ± 0.58		2.91 ± 0.65	2.91 ± 0.65	0.55 ± 0.97	
Anorexia	Non intervention	12	86.50 ± 10.93	81.83 ± 13.33	−4.67 ± 8.37	0.582	4.67 ± 0.65	4.00 ± 0.81	− 0.67 ± 0.64	0.674	4.36 ± 0.53	3.99 ± 0.69	− 0.38 ± 0.62	0.539	3.93 ± 0.57	3.63 ± 0.85	− 0.30 ± 0.62	0.180	1.92 ± 0.68	2.48 ± 0.42	0.57 ± 0.64	0.923
	Intervention	10	88.70 ± 5.54	86.60 ± 9.92	−2.10 ± 7.43		4.93 ± 0.16	4.35 ± 0.73	− 0.58 ± 0.67		4.33 ± 0.53	4.08 ± 0.58	− 0.25 ± 0.69		3.74 ± 0.43	3.72 ± 0.56	− 0.02 ± 0.70		2.24 ± 0.82	2.76 ± 0.56	0.52 ± 1.02	
Peripheral sensory neuropathy	Non intervention	4	83.25 ± 15.74	81.25 ± 15.84	−2.00 ± 3.46	1.000	4.42 ± 0.79	3.67 ± 1.03	−0.75 ± 0.52	0.343	4.21 ± 0.88	4.21 ± 0.60	0.00 ± 0.30	0.343	3.75 ± 0.66	3.60 ± 1.02	−0.15 ± 0.62	0.686	1.95 ± 0.79	2.55 ± 0.25	0.60 ± 0.95	0.686
	Intervention	4	86.50 ± 14.93	81.50 ± 12.66	−5.00 ± 10.39		4.38 ± 1.25	4.08 ± 1.02	− 0.29 ± 0.72		4.33 ± 0.14	4.08 ± 0.52	−0.25 ± 0.40		3.85 ± 0.76	3.40 ± 0.57	− 0.45 ± 0.85		2.35 ± 1.10	2.45 ± 0.55	0.10 ± 0.62	
Alopecia	Non intervention	19	88.16 ± 13.40	85.26 ± 15.17	−2.89 ± 7.49	0.603	4.61 ± 0.66	4.21 ± 0.77	−0.40 ± 0.73	0.452	4.33 ± 0.69	4.07 ± 0.74	− 0.26 ± 0.53	0.665	3.94 ± 0.67	3.75 ± 0.87	− 0.19 ± 0.55	0.525	2.32 ± 0.91	2.68 ± 0.71	0.37 ± 0.62	0.563
	Intervention	19	91.74 ± 9.92	88.37 ± 11.14	−3.37 ± 7.63		4.79 ± 0.58	4.22 ± 0.74	−0.57 ± 0.60		4.43 ± 0.51	4.12 ± 0.48	−0.31 ± 0.53		4.05 ± 0.66	3.92 ± 0.63	− 0.14 ± 0.67		2.50 ± 0.83	3.00 ± 0.68	0.51 ± 0.91	
Nail ridging	Non intervention	11	89.36 ± 11.53	86.91 ± 14.00	−2.45 ± 7.57	0.494	4.76 ± 0.52	4.21 ± 0.68	−0.55 ± 0.69	0.392	4.33 ± 0.73	4.21 ± 0.69	−0.12 ± 0.36	0.303	3.98 ± 0.75	3.87 ± 0.86	− 0.11 ± 0.40	0.733	2.27 ± 0.79	2.66 ± 0.62	0.38 ± 0.62	0.303
	Intervention	13	91.92 ± 8.39	87.92 ± 10.70	−4.00 ± 8.02		4.88 ± 0.73	4.17 ± 0.65	−0.72 ± 0.63		4.45 ± 0.57	4.08 ± 0.56	−0.37 ± 0.59		4.14 ± 0.68	3.89 ± 0.66	− 0.25 ± 0.70		2.31 ± 0.83	3.08 ± 0.56	0.77 ± 0.83	
Skin disorder	Non intervention	8	91.00 ± 14.69	87.50 ± 17.18	−3.50 ± 8.00	0.442	4.75 ± 0.58	4.10 ± 1.01	−0.65 ± 0.76	0.382	4.42 ± 0.67	4.13 ± 0.62	−0.29 ± 0.50	1.000	4.25 ± 0.62	4.00 ± 0.89	− 0.25 ± 0.49	0.234	2.23 ± 1.17	2.93 ± 0.76	0.70 ± 0.58	0.645
	Intervention	8	89.63 ± 7.19	84.88 ± 9.16	−4.75 ± 7.13		4.90 ± 0.18	4.06 ± 0.68	−0.83 ± 0.62		4.31 ± 0.57	3.94 ± 0.53	−0.38 ± 0.74		3.78 ± 0.33	3.80 ± 0.60	0.03 ± 0.59		2.43 ± 0.87	2.88 ± 0.62	0.45 ± 1.09	

Mann-Whitney’s U-test, mean ± standard deviation, * *P* < 0.05

Table 4 shows the difference between immediately before the 1st course and immediately before the 2nd course. The intergroup comparisons of the subscale of social relationships showed a significant difference in malaise (0.50 ±0.66 and 1.24 ±0.85; *p* = 0.043, respectively) with the non-intervention group experiencing it to a greater degree than the intervention group.

Table 5 shows the difference between immediately before the 1st course and immediately before the 3rd course. The intergroup comparisons of the subscale of social relationships (−0.46 ±0.67 and 0.60 ±0.20; *p* = 0.006, respectively) showed a significant difference regarding nausea with the non-intervention group experiencing it to a greater degree than the intervention group.

## Discussion

In the present study, we investigated the impact of pharmacist counseling on the QOL of outpatients with breast cancer receiving chemotherapy. We determined this impact by applying QOL-ACD questionnaires. Concurrently, we investigated adverse events caused by treatment. This allowed us to evaluate not only the overall impact of adverse events related to outpatient chemotherapy but also the individual impact of each adverse event. The patients involved in the study were suitably representative of the wider population, because the attributes of each were similar to those of patients reported in research by the Japanese Breast Cancer Society. As there was no statistically significant difference found between the non-intervention group and the intervention group, in relation to attributes, they were deemed similar in this regard.

According to the mean scores for the QOL-ACD subscales of activity and physical condition, significant reductions in QOL occurred in both groups after outpatient chemotherapy; however, no significant decrease in the total QOL-ACD score was observed. The significant decrease observed in the mean scores of the QOL-ACD subscales suggests that adverse events did not affect overall QOL, but that adverse events contribute to decreasing individual QOL in the home and workplace. This indicates that adverse events caused by anticancer drugs not only interfere with patients’ activities but also exacerbate their physical states. On the other hand, the mean score for the QOL-ACD subscale concerning social relationships increased significantly. The social relationships subscale comprises items relating to anxiety and relationships with family and friends. Almost all the studied patients lived with family; therefore, the social aspects of their QOL may have improved because of family support.

The QOL scores of the non-intervention group and the intervention group were compared to examine the overall effects of the patients who reported experiencing adverse events. Comparing the score between immediately before the 1st course and immediately before the 2nd course shows that a significant difference arose the subscale of social relationships regarding malaise. Malaise is common among patients with cancer who have received cancer chemotherapy and it causes substantial adverse physical and psychosocial effects for both patients and caregivers [26]. Malaise caused by cancer chemotherapy has a strong cause-and-effect relationship with patients’ QOL [27]. The subscales of social relationships for malaise was lower in the non-intervention group than in the intervention group at the before 2nd course. Instances of malaise caused by cancer chemotherapy can be lessened through the active intervention of medical staff, as they can assess malaise, provide psychological support, and coach patients on self-care. These interventions enable patients to adapt to living with malaise and can help them improve their psychological/emotional well-being and ability to cope with their illness and treatment [28]. In addition to the guidance provided by attending physicians and nurses, pharmacists also participated in patient guidance by providing counseling, and this is believed to explain why the influence of intervention was stronger in the intervention group than in the non-intervention group.

In the comparisons between immediately before the 1st course and immediately before the 3rd course, a significant difference was noted in the subscale of psychological condition regarding nausea. Before the 2nd course, a decrease in QOL score was observed in both groups; however, in the intervention group an increase in QOL score was also observed before the 3rd course. Nausea was reported to reduce QOL to a much greater degree than other adverse events [29]. For the intervention group, during the counselling session held after the beginning of chemotherapy the pharmacist listened to patients’ descriptions of adverse events and then proposed supportive therapy based on analysis of the events. As descriptions of adverse events vary greatly depending on the patient, the 1st course was treated with general supportive therapy; however, the 2nd course involved individually adjusted supportive therapy based on patient’s description of the adverse events experienced during the 1st course. When the individual supportive therapy was shown to be effective against adverse events that occurred during the 2nd course and later, we considered the adverse events to be reduced. In the intervention group, the pharmacist interviewed participants on adverse events, including nausea, and then considered countermeasures tailored to suit the adverse events experienced by each patient. Regarding the symptoms of nausea, since guidelines are being developed for other supportive therapies it is easy to use information on these events to create effective treatment proposals. Regarding using medicine to treat nausea and vomiting, the pharmacist in our study was an expert in medicinal drugs and understood the characteristics and dynamics of each medicine in detail. Therefore, it was possible to make an appropriate prescription recommendation for each patient. The pharmacist proposed that the attending physician should implement antiemetic measures customized to suit each patients’ experience of adverse events. These measures were implemented and were considered to reduce the occurrence of nausea, which in turn caused an improvement in the QOL of the intervention group.

Alopecia and anorexia were side effects that occurred in more than half of patients, immediately before the 2nd course and immediately before the 3rd course, respectively. In previous qualitative studies, women reported that alopecia is associated with a loss of privacy because it makes the environment aware that the person is receiving chemotherapy [30]. It is also a visible reminder of the disease [31] and signals the seriousness of cancer [32]. Consequently, alopecia has been reported to be associated with lower QOL [33]. In addition, in our research, alopecia occurred in most patients, and in patients who showed depilation, both groups showed a decline in total QOL, activity, physical condition, and psychological condition. Since there is no fundamental countermeasure for alopecia caused by cancer chemotherapy and it is not possible to deal with medicine, even if there is involvement of a pharmacist, the side effects may negatively affect the non-intervention group’s QOL.

Anorexia was another side effect that occurred in many patients. There is no drug to remedy anorexia; therefore, the only clear way to reduce anorexia is by decreasing the dose of anticancer drugs that are causing anorexia. However, because there are few drugs that anorexia as a dose limited factor of anticancer drugs, in actual clinical practice, we often respond by dealing with dietary intake adjustment and ingestion method as coping mechanisms. Since a pharmacist is involved, patients can be educated about how to deal with anorexia; however, but since anorexia itself does not disappear, the rate of anorexia was similar in both groups. Therefore, there was no difference in QOL.

We found that pharmacists can improve chemotherapy patients’ QOL regarding malaise and nausea by providing personal counseling before the medical examinations. In addition to the attending physician and nurses, pharmacists can also partially alleviate malaise through active intervention that involves patient counseling and guidance. Previous reports have stated that improvements in symptoms and reductions in adverse events after pharmacists’ interventions were assisted by the care provided by the medical team, including pharmacists [13]. However, this fails to consider the fact that, in outpatient cancer chemotherapy, patients are away from the hospital for a time after treatment and, consequently, the medical staff cannot determine patients’ condition. When a patient visits a hospital for treatment, the medical staff can provide patient care, addressing various aspects by conducting patient counseling and listening to patients’ descriptions of adverse events; however, pharmacists can give prescription suggestions based on pharmacokinetics and drug properties; therefore, this supportive-therapy method allows patients to alleviate adverse events in the field, where there are abundant drug therapy options.

In the present study, we used QOL-ACD questionnaires to assess the impact of pharmacist counseling on adverse events that affect the QOL of chemotherapy outpatients with breast cancer; however, our study has several limitations; for example, its small sample size, being conducted at a single institution, and a lack of other relevant clinical information on the influence of multiple- and lower-level adverse events on QOL. Despite these limitations, our findings reveal that, through personal counseling, pharmacists can improve chemotherapy outpatients’ QOL regarding malaise and nausea.

## Conclusions

Although patients’QOL decreases when receiving cancer chemotherapy, it is desirable that pharmacists support the treatment by providing guidance, listening to patients who have undergone chemotherapy, and collaborating with other medical staff to maintain patients’ QOL. We believe that our study findings are very useful and important for patients, as this constitutes a means of maintaining QOL during cancer treatment.

## Abbreviations

CTCAE: The Common Terminology Criteria for Adverse Events, version 4.0
ER: Estrogen receptor
HER2: Human epidermal growth factor receptor type 2
PgR: Progesterone receptor
PS: Performance status
QOL: Quality-of-life
QOL-ACD: The Quality of Life Questionnaire for Cancer Patients Treated with Anticancer Drugs
SPSS: The IBM Statistical Package for Social Science
